# Oral and Intravenous Fumonisin Exposure in Pigs—A Single-Dose Treatment Experiment Evaluating Toxicokinetics and Detoxification

**DOI:** 10.3390/toxins10040150

**Published:** 2018-04-05

**Authors:** Hanna Schertz, Jeannette Kluess, Jana Frahm, Dian Schatzmayr, Ilse Dohnal, Gerlinde Bichl, Heidi Schwartz-Zimmermann, Gerhard Breves, Sven Dänicke

**Affiliations:** 1 Friedrich-Loeffler-Institute, Federal Research Institute for Animal Health, 38116 Braunschweig, Germany; hanna.schertz@fli.de (H.S.); jana.frahm@fli.de (J.F.); sven.daenicke@fli.de (S.D.); 2 BIOMIN Holding GmbH, BIOMIN Research Center, 3430 Tulln, Austria; dian.schatzmayr@biomin.net (D.S.); ilse.dohnal@biomin.net (I.D.); gerlinde.bichl@biomin.net (G.B.); 3 Christian Doppler Laboratory for Mycotoxin Metabolism, IFA, 3430 Tulln, BOKU Vienna, Austria; heidi.schwartz@biomin.net; 4 Institute for Physiology, University of Veterinary Medicine Hanover, Foundation, 30559 Hanover, Germany; gerhard.breves@tiho-hannover.de

**Keywords:** fumonisin, pigs, toxicokinetic, single-dose, fumonisin esterase, degradation, mycotoxin

## Abstract

We examined the toxicokinetics of fumonisin B_1_ (FB1) and its main metabolites after single dose application *intravenously* (*iv*) of 139 nmol FB1 or hydrolyzed FB1 (HFB1)/kg bodyweight (BW) in barrows (BW: 34.4 kg ± 2.7 kg), as well as the toxicokinetics of FB1, FB2, FB3 and FB1 bioavailability from oral exposure (3425 nmol FB1/kg BW, on top of ration). Additionally, detoxification efficacy of FumD (240 U/kg feed; 3321 nmol FB1/kg BW), a fumonisin esterase, was examined for oral fumonisin application. Urine and feces were collected quantitatively and serum samples were taken over a period of 120 h. Serum toxicokinetics of FB1*iv* showed a short distribution half-life of 6 min followed by a longer elimination half-life of 36 min. After HFB1*iv* administration, serum clearance was three times higher compared to FB1*iv* group (5.6 and 1.8 L/kg/h respectively) which together with a 5-times higher volume of distribution indicates that HFB1 is more rapidly cleared from systemic circulation but distributed more extensively into the extravasal space than FB1. The bioavailability of FB1 in orally exposed pigs was 5.2% (incl. metabolites). Moreover, we found a significant reduction of FB1 bioavailability by 90% caused by the action of fumonisin esterase in the gastrointestinal tract, clearly demonstrating the efficacy of FumD.

## 1. Introduction

The Food and Agricultural Organization (FAO) reported that approximately 25% of the world’s agricultural commodities are contaminated with mycotoxins which leads to significant economic losses [1]. Fumonisins, an important group of mycotoxins, were first isolated in 1988 from a culture of *Fusarium verticillioides* strain [2]. Animal and human health problems related to these mycotoxins are almost exclusively associated with the consumption of contaminated maize and feed made from maize [3]. More than 28 fumonisin homologues were discovered in the last decades but fumonisin B1 (FB1) is one of the main contaminants of animal feed worldwide [3]. Therefore, most toxicological studies focus on FB1. FB2, FB3 and FB4 differ structurally from FB1 and are less prevalent [4]. However, co-occurrence of FB1 with several other mycotoxins- fumonisins (main categories are A, B, C and P) as well as other classes of mycotoxins, are not only possible but rather likely [5]. FB1 predominantly causes leukoencephalomalacia in horses (ELEM, [6]), hepatotoxicity in rats [7], pulmonary edema in swine (PPE, [8]) and alterations of the immune response in pigs [9]. Moreover, FB1 is known to be carcinogenic and is associated with esophageal cancer and neural tube disorder in humans [10]. On account of these adverse health effects both in animals and humans, guidance levels for fumonisins were established by the European Commission whereby the recommended maximum FB1+FB2 concentration in swine and horse feed must not exceed 5 mg/kg feed and 60 mg/kg in maize and maize products [11]. An acute trigger dose of <4.5 mg/kg bodyweight (BW) was implemented by Scientific Committee on Food for the onset of PPE [12], which is linked to hydrothorax, alterations in heart and circulatory system and death in pigs within days [13]. Despite of its poor bioavailability in all investigated species, FB1 is well known to cause various toxicological effects, which are referred to as the “fumonisin paradox” [14]. Certainly, the toxin concentration in blood, organs, urine and feces is influenced by the metabolism of the toxin to a large extent and only limited data is reported in literature about the metabolism of FB1 so far [4]. It is known that the liver and kidneys are primarily responsible for the metabolism and excretion of fumonisin and that enterohepatic recycling extends the residence time within the organism [15]. Toxicological effects are associated with an interference of fumonisin with sphingolipid metabolism by inhibiting the ceramide synthase (CerS), a central enzyme of *de novo* ceramide synthesis [4,16]. An alteration in sphingoid base proportion is responsible for the toxic and carcinogenic effects of fumonisins [17,18,19]. 

Among others, a suitable post-harvest decontamination method for FB1 in feed is detoxification by an enzyme with esterase activity [20]. FumD, a type-B carboxylesterase, was reported to detoxify FB1 by hydrolysis of both tricarballylic acid (TCA) side chains. The resulting hydrolyzed form, HFB1, was proven to have a strongly reduced toxicity compared to FB1 in pigs [21,22]. The toxicity of the intermediate, partially hydrolyzed FB1 (pHFB1), has not been comprehensively investigated so far. In the only published *in vivo* study pHFB1 has been shown to be non-toxic in rats [23]. In a recent study, it was shown that after oral (*per os* = *po*) exposure of pigs to 2 mg FB1+FB2/kg feed and application of 60 U FumD/kg feed (FUM*zyme*^®^, BIOMIN GmbH, Tulln, Austria), the feed additive was effective in preventing the fumonisin-associated disruption of sphingolipid metabolism [22]. FumD is a fumonisin esterase that was isolated from soil bacteria *Sphygopyxis* sp. MTA 144. After elucidating the catabolic pathway and identifying the gene cluster encoding enzymes for FB1 degradation, FumD is the first-ever purified enzyme proven to transform fumonisin into a hydrolysis product (HFB1) [22].

In previous studies, the biological fate and the toxicokinetics of fumonisins has not yet been fully elucidated in pigs [13,24,25,26], either providing the toxin chronically or lacking frequent blood sampling necessary for toxicokinetic evaluation. Therefore, in the present study a single-dose experiment was conducted evaluating the toxicokinetics and bioavailability of FB1 and HFB1 as well as the biological fate and excretion of FB1, FB2 and FB3. Furthermore, the latter was also investigated in absence and presence of a fumonisin esterase (FumD) in order to study the efficacy of the enzymatic detoxification of fumonisin *in vivo*. 

## 2. Results

### 2.1. Toxicokinetic Study

#### 2.1.1. Serum Toxicokinetics of the FB1 Group Dosed Intravenously (FB1*iv*)

**Figure 1:**
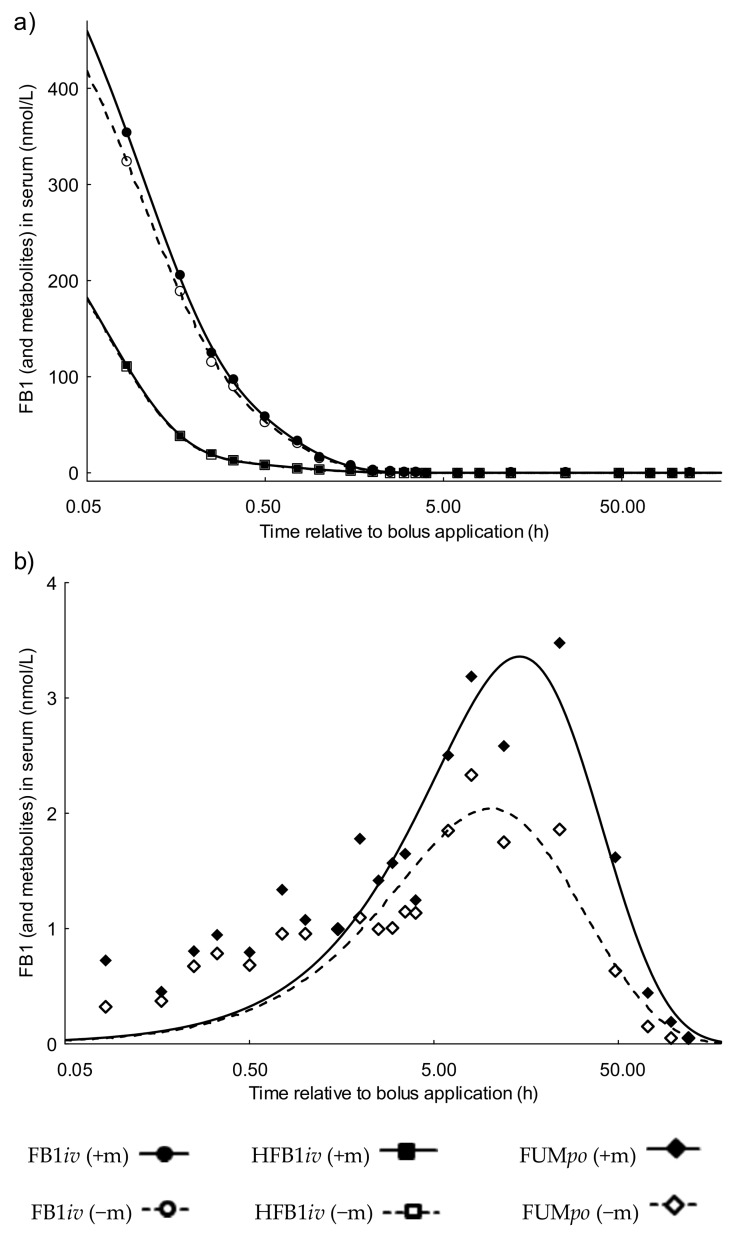
Plots of serum kinetics (derived from regression analysis) of fumonisin B_1_ (FB1−m) (FB1 alone) or FB1+m (sum of FB1 and metabolites: hydrolyzed FB1 (HFB1), pHFB1a and pHFB1b) in group FB1*iv* and Fumonisin Group Dosed Orally (FUM*po*) or HFB1−m (HFB1 alone) or HFB1+m (HFB1 and the sum of metabolites: pHFB1a and pHFB1b) in group HFB1*iv* as a function of time (semi-logarithmic plot) after bolus treatment. Panel (**a**) FB1*iv* and HFB1*iv* and Panel (**b**) FUM*po* treated pigs. Pigs were randomly allocated to the different groups and treated with: FB1*iv*: 139 nmol·kg·BW^−1^; HFB1*iv*: 139 nmol·kg·BW^−1^ and FUM*po*: 3377 nmol FB1·kg·BW^−1^ + 1367 nmol FB2·kg·BW^−1^ + 584 nmol FB3·kg·BW^−1^. Frequent serum samples were taken over a period of 120 h post toxin application (*n* = 6/group).

The results for FB1 alone (FB1−m) or FB1 plus metabolites (FB1+m; m = HFB1, pHFB1a and pHFB1b) are shown in Figure 1 panel (a), depicting the typical time course after *iv* substance application: In FB1*iv* group, initial distribution was rapid with a mean half-life (t_1/2α_) of 0.08 ± 0.03 h, followed by a slower elimination phase (t_1/2β_) of 0.57 ± 0.4 h (Table 1) for FB1−m. The apparent volume of distribution of FB1 was higher than the total body water (V_d_ = 1.8 ± 2.1 L/kg) for FB1−m (Table 1). The serum clearance (Cl) of FB1−m was 1.8 ± 1.1 L/kg BW/h. 

Toxicokinetic parameters of FB1+m did not or just marginally differ from FB1−m for t_1/2α_, t_1/2β_, V_d_ and Cl. In FB1*iv* group, AUC for FB1−m in serum compared to FB1+m was 93% ± 3%, whereby AUC for FB1+m and FB1−m were 118 ± 86 and 129 ± 96 nmol·L^−1^·h, respectively (Table 1). 

#### 2.1.2. Serum Toxicokinetics of the Hydrolyzed FB1 Group Dosed Intravenously (HFB1*iv*)

The *iv* toxicokinetics of completely hydrolyzed fumonisin B1 (HFB1) which is the main product of enzymatic hydrolysis of FB1 were also determined. The results for HFB1 alone (HFB1−m) or HFB1 plus metabolites (HFB1+m; m = pHFB1a and pHFB1b) are shown in Figure 1 panel (a). The kinetic profile was similar to that observed for FB1 (Table 2). The mean half-life of distribution in HFB1*iv* group was even shorter than for FB1*iv* group with t_1/2α_ = 0.05 ± 0.02 h for HFB1−m. The elimination phase t_1/2β_ was 1.01 ± 0.8 h for HFB1−m. The apparent volume of distribution of hydrolyzed fumonisin in the HFB1*iv* group was higher than that for FB1 in FB1*iv* group with V_d_ = 11.0 ± 10.3 L/kg for HFB1 alone. The serum clearance (Cl) was 6.7 ± 3.2 L/kg BW/h for HFB1−m. 

Parameters of HFB1+m were slightly different with t_1/2α_ = 0.06 ± 0.02 h, t_1/2β_= 1.20 ± 0.85 h, V_d_ = 12.4 ± 10.7 L/kg, Cl = 6.4 ± 3.2 L/kg BW/h. The AUC for HFB1−m in serum compared to HFB1+m was 96.1% ± 3.9%, whereby AUC for HFB1+m and HFB1−m were 30 ± 27 and 32 ± 28 nmol·L^−1^·h, respectively (Table 2).

#### 2.1.3. Serum Toxicokinetics of the Fumonisin Group Dosed Orally (FUM*po*)

The *po* kinetics are characterized by an initial increase, preceding a peak, which is followed by a decrease (Figure 1 panel (b)). Moreover, concentrations are differing between FB1−m and FB1+m, the latter reaching considerably higher concentrations. As described in Table 3, FB1 was very slowly absorbed with a mean invasion half-life of t_1/2ka_ = 4.2 ± 3.7 h for FB1−m and 6.1 ± 2.8 h for the FB1+m. Peak concentrations (C_max_) of 2.0 ± 0.5 nmol FB1−m or 3.3 ± 1.7 nmol FB1+m per L serum were reached (t_max_) after 9.5 ± 5.4 h and 13.6 ± 2.5 h, respectively. The mean elimination half-life t_1/2ke_ was 22.8 ± 125.5 h for FB1−m and 20.7 ± 10.2 h for FB1+m which was significantly slower for FB1−m (*p* = 0.001). For FB1−m, the apparent volume of distribution was V_d_ = 8.7 ± 2.1 L/kg BW while Cl = 0.4 ± 0.1 L/kg BW·h. For FB1+m, V_d_ was 7.7 ± 1.8 L/kg BW however Cl was the same as for FB1−m, 0.4 ± 0.3 L/kg BW·h. V_d_ for FB1 (*p* = 0.014) and metabolites (*p* = 0.043) was significantly higher than in *iv* groups and Cl was lower (FB1−m: *p* = 0.042; FB1+m: *p* = 0.049). The mean residence time of toxin in the invaded compartment (MRT) was 25.4 ± 6.9 h for FB1−m but not significantly higher for FB1+m (28.6 ± 5.4 h). The AUC for FB1−m in serum compared to FB1+m was 62.6% ± 20.3% in FUM*po* group (Table 3). AUC% was significantly lower for FB1+m in FUM*po* group than in FB1*iv* group (*p* = 0.006).

FB2, FB3 and their respective metabolites’ concentrations in serum were <LOQ (Table 7) and therefore could not be evaluated. 

#### 2.1.4. Bioavailability

The mean bioavailability of FB1 after *po* exposure was low: for FB1−m it amounted to F_AUC_ = 3.1% ± 0.4 with which it was somewhat higher for FB1+m with F_AUC_ = 5.2% ± 1.3 (Table 3).

### 2.2. Comparative Fumonisin Kinetics after Oral Administration in the Absence (FUMpo) and Presence of Dietary Fumonisin Esterase (FumDpo)

**Figure 2:**
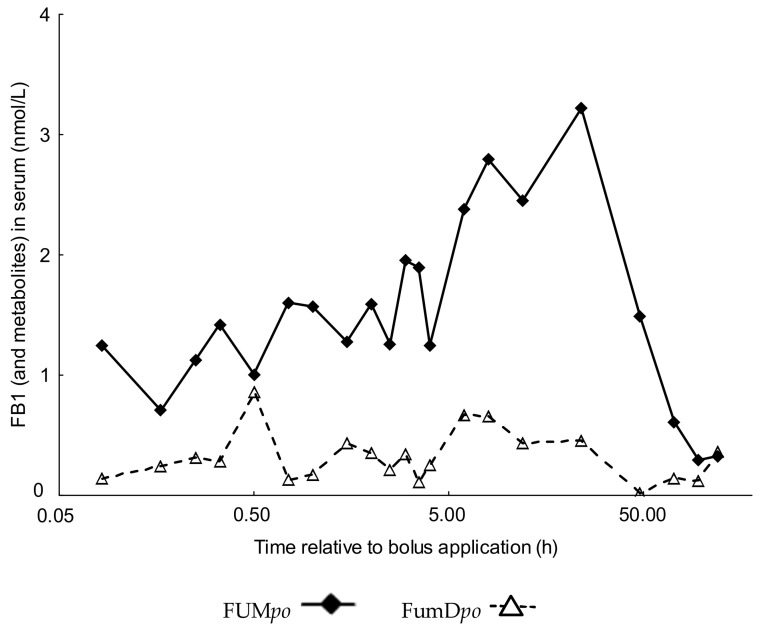
FB1 and metabolites toxicokinetics in *po* treated pigs with or without fumonisin esterase (+/−FumD). Plot of serum mean concentrations (nmol/L; y-axis) of FB1 and metabolites (HFB1, pHFB1a and pHFB1b) and time (x-axis; semi-logarithmic plot) as means of the particular groups (*n* = 6/group). Pigs were randomly allocated to the different groups and treated as follows: FUM*po*: 120 mg FB1 + 48 mg FB2 + 14 mg FB3/kg feed or FumD*po*: 120 mg FB1 + 48 mg FB2 + 14 mg FB3/kg feed + 240 U FumD/kg feed. Frequent serum samples were taken over a period of 120 h post toxin application.

As blood levels of FB1 and metabolites did not follow the typical course of absorption up to a peak level followed by a decrease characterizing the elimination phase when the fumonisin-containing diet was supplemented with the esterase, a kinetic evaluation of the data could not be performed. Instead, blood toxin residue levels were compared between FUM*po* and FumD*po* for each sampling time (Figure 2). First significant differences in FB1 and metabolite concentrations occurred 45 min after toxin application (*p* = 0.008). Further significant differences were detected at 1 h, 2.5 h, 6 h, 8 h, 12 h, 24 h and 48 h after dosage. From 72 to 120 h after toxin administration, no significant differences between groups could be detected any more.

Due to the absence of a serum kinetic data suitable for regressive evaluation of AUC for group FumD*po*, it was quantified by the trapezoidal method and used for further calculation of bioavailability. Significant differences were found both in AUC (*p* = 0.01) and bioavailability F_AUC_ (*p* = 0.01, Table 4) whereby AUC and F_AUC_ were both significantly lower in FumD*po* group compared to FUM*po* group both for FB1+m and FB1−m. Decreases in AUC of 97.6% and in bioavailability of 96.8% after enzyme application for FB1−m and a reduction in AUC of 83.5% and in bioavailability of 84.6% for FB1+m, respectively were determined.

### 2.3. Urinary and Fecal Excretion and Metabolite Profiles 

**Figure 3:**
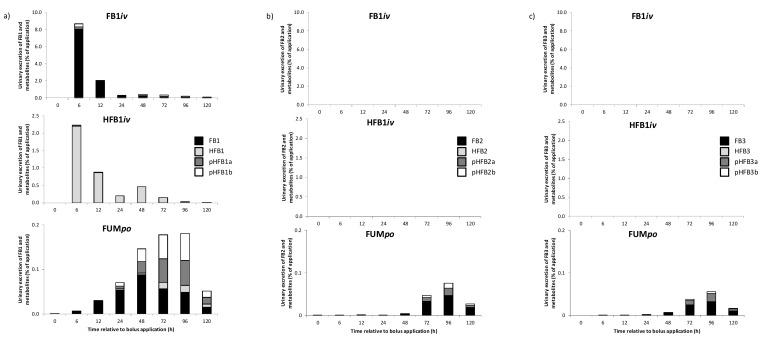
Metabolite profile of fractional urinary excretion of fumonisins and their respective metabolites in percentage of toxin application at different time points for groups FB1*iv* (139 nmol·kg·BW^−1^), HFB1*iv* (139 nmol·kg·BW^−1^) and FUM*po* (3377 nmol·kg·BW^−1^ FB1 + 1367 nmol·kg·BW^−1^ FB2 + 584 nmol FB3·kg·BW^−1^) over a sampling period of 120 h (means of *n* = 6/group). Quantitative collection was performed 12 h before and 6, 12, 24, 48, 72, 96 and 120 h post toxin application. (**a**) FB1, HFB1, pHFB1a, pHFB1b main effects (*F*-test): *p*_group_ < 0.001, *p*_time_ < 0.001; *p*_group*time_ < 0.001 for all substances. (**b**) FB2, HFB2, pHFB2a, pHFB2b main effects (*F*-test): *p*_group_ < 0.001, *p*_time_ < 0.001; *p*_group*time_ < 0.001 for all substances. (**c**) FB3, HFB3, pHFB3a, pHFB3b main effects (*F*-test): p_group_ < 0.001, p_time_ < 0.001; p_group*time_ < 0.001 for all substances.

Urinary excretion patterns (% of application) of FB1, FB2 and FB3, including their metabolites, are depicted in Figure 3 for groups FB1*iv*, HFB1*iv* and FUM*po*. 

The peak of fractional toxin excretion in urine in group FB1*iv* was detected already after 6 h (~8%), mainly consisting of paternal FB1 (Figure 3) and decreased markedly afterwards. The same pattern was detectable for group HFB1*iv*, where peak excretion of the paternal HFB1 was also reached after 6 h (~2%) and again only negligent levels of metabolites were observed. Neither FB2 and FB3 nor their respective metabolites were detected in urine after *iv* administration of FB1 and HFB1. 

Excretion pattern in group FUM*po* with oral toxin exposure stood in stark contrast to *iv* administration, reaching peak excretion only after 96 h (<1%) and more than 50% comprising of metabolites, mainly the partially hydrolyzed fumonisins. Furthermore, FB2, FB3 and their metabolites showed similar excretion characteristics in group FUM*po*, albeit to a lesser extent than FB1, amounting to less than 50% of FB1 excretion levels (Figure 3). 

The cumulative urine excretion 120 h after toxin bolus administration is detailed in Table 5. Group FB1*iv* showed significantly higher cumulative excretion of FB1−m and FB1+m, compared to HFB1*iv* and both oral groups, FUM*po* and FumD*po*. The main excretory route for *iv* administered FB1 was the urinary route with ~12% of application, whereas group HFB1*iv* only showed ~ 4% excretion via urine (HFB1 and metabolites) and expectedly no FB1 excretion at all (FB1 –m). Both oral groups, with (FumD*po*) or without (FUM*po*) esterase supplementation, excreted less than 1% via urine. Albeit excreted at very low levels, FB1 alone was eliminated significantly less in group FumD*po* compared to FUM*po* (0.012 vs. 0.24%), although this could not be verified statistically for FB1+m. Even lower cumulative urinary excretions were calculated for FB2 and FB3 (−/+m), amounting to less than 0.1% of exposure and displaying no differences between FUM*po* and FumD*po* (S2).

The second excretory route measured was those via feces, also provided in Table 5. Proportions were basically reversed to urinary elimination: highest fecal excretion of FB1 was determined for oral group FUM*po* with ~25% excretion and less than 2% for FumD*po* and *iv*-groups. Interestingly, the drastic reduction in FB1-excretion for group FumD*po* was not reflected in FB1+m, indicating a shift to fumonisin metabolites due to esterase inclusion into the animal ration. Also, in group FB1*iv* there was still substantial metabolism of FB1 in feces, as FB1+m was determined at 9% of application in contrast to only ~2% FB1−m. Fecal excretion was also the main route for FB2, FB3 and their respective metabolites and here statistical analyses revealed that excretion was always significantly lower in group FumD*po* as compared to FUM*po* (S2). Metabolite production was not as extensive as for FB1 and here as well there was significantly less excreted in FumD*po*.

With regard to the fractional excretion pattern (Figure 3), ~1.4% (48 h) and ~0.6% (72 h) FB1, ~2.7% (48 h) and ~1.3% (72 h) pHFB1a and ~1.5% (48 h) and ~0.7% (72 h) pHFB1b were detected in feces in the FB1*iv* group but no HFB1 could be found. In HFB1*iv* group, FB1 was recovered once after 48 h. In contrast to urinary excretion, FB1 and HFB1 in HFB1*iv* group were detected in feces after 48 h and 48 and 72 h, respectively. In addition, no partially hydrolyzed fumonisin could be detected in feces. 

**Figure 4:**
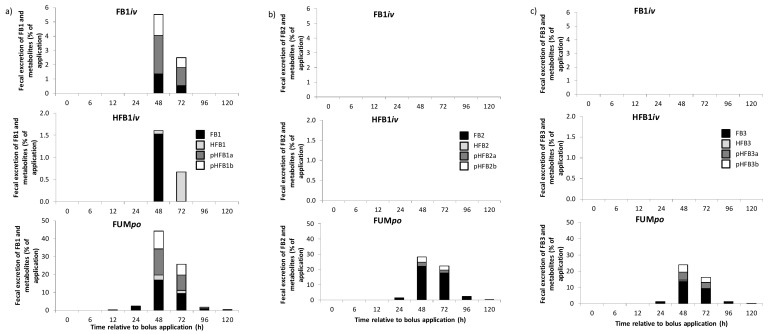
Metabolite profile of fractional fecal excretion of fumonisins and their respective metabolites in percentage of toxin application at different time points for groups FB1*iv* (139 nmol·kg·BW^−1^), HFB1*iv* (139 nmol·kg·BW^−1^) and FUM*po* (3377 nmol·kg·BW^−1^ FB1 + 1367 nmol·kg·BW^−1^ FB2 + 584 nmol FB3·kg·BW^−1^) over a sampling period of 120 h (means of *n* = 6/group). Quantitative collection was performed 12 h before and 6, 12, 24, 48, 72, 96 and 120 h post toxin application. (**a**) FB1, HFB1, pHFB1a, pHFB1b main effects (*F*-test): *p*_group_ < 0.001, *p*_time_ < 0.001; *p*_group*time_ < 0.001 for all substances. (**b**) FB2, HFB2, pHFB2a, pHFB2b main effects (*F*-test): *p*_group_ < 0.001, *p*_time_ < 0.001; *p*_group*time_ < 0.001 for all substances. (**c**) FB3, HFB3, pHFB3a, pHFB3b main effects (*F*-test): *p*_group_ < 0.001, *p*_time_ < 0.001; *p*_group*time_ < 0.001 for all substances.

In FUM*po* group, all degradation products were found in feces as early as 12 h after the bolus with a peak after 48 h. Compared to urinary excretion (~1%) and *iv* dosed pigs, much higher amounts were excreted via the fecal route in FUM*po* group (~45%, peak) with FB1 being the predominant toxin. Partially hydrolyzed forms (pHFBa, pHFBb) were found in similar proportions and only small amounts of completely hydrolyzed FB1 were recovered. For FB2 and FB3 excretion a kinetic pattern similar to that of FB1 was found. However, more parent toxin and less metabolites were excreted. Neither FB2, FB3 nor their metabolites were detected in feces after *iv* administration of FB1 and HFB1. The highest excretion was revealed in FUM*po* group also with peak excretions at 48 h and varying metabolite proportions (Figure 4). 

**Figure 5:**
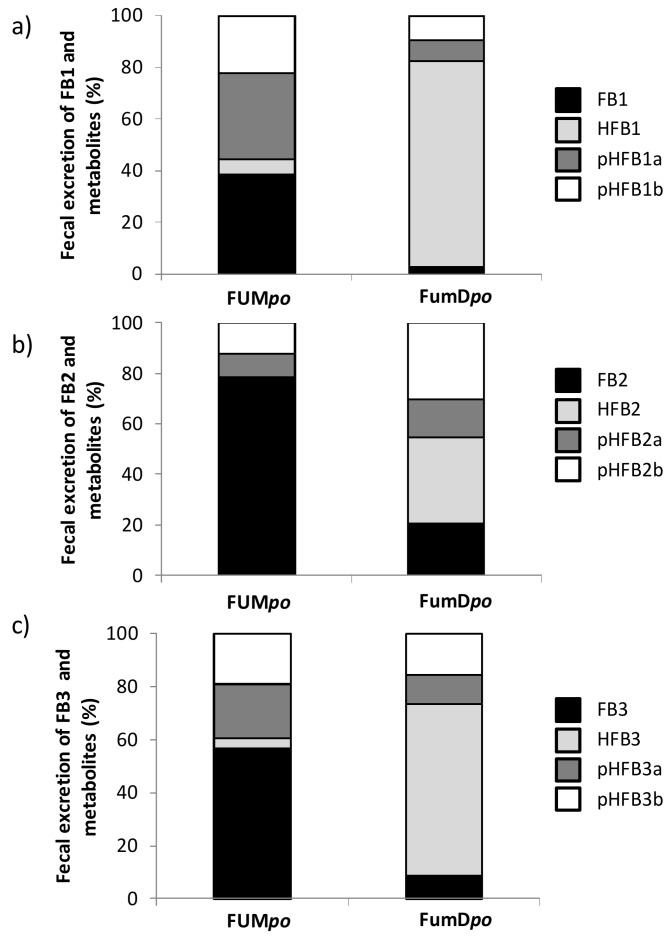
Metabolite profiles of fractional fecal excretion of fumonisins and their metabolites expressed as percentage of the sum of all toxins (means of *n* = 6/group) at 48 h post toxin application for group FUM*po* (120 mg FB1 + 48 mg FB2 + 14 mg FB3/kg diet) and FumD*po* (120 mg FB1 + 48 mg FB2 + 14 mg FB3/kg diet and 240 U fumonisin esterase per kg diet). (**a**) FB1 and metabolites (HFB1, pHFB1a, pHFB1b) with following main effects (*F*-test): *p*_FB1_ = 0.009, *p*_HFB1_ = 0.002; *p*_pHFB1a_ = 0.0260; *p*_pHFB1b_ = 0.026. (**b**) FB2 and metabolites (HFB2, pHFB2a, pHFB2b) with following main effects (*F*-test): *p*_FB2_ = 0.015, *p*_HFB2_ = 0.002; *p*_pHFB2a_ = 0.240; *p*_pHFB2b_ = 0.937. (**c**) FB3 and metabolites (HFB3, pHFB3a, pHFB3b) with following main effects (*F*-test): *p*_FB3_ = 0.015, *p*_HFB3_ = 0.065; *p*_pHFB3a_ = 0.041; *p*_pHFB3b_ = 0.093.

In the previous section, cumulative urinary and fecal excretion of FB1−m and FB1+m was already provided in Table 5. A significantly lower excretion of FB1−m via urine and feces was detected after esterase administration (FumD*po* group) compared to FUM*po* but not for FB1+m, reflecting the amount of metabolism achieved for FumD*po*. For further illustration, metabolite profiles of fractional fecal excretion are depicted in Figure 5, representing 48 h post toxin application as the peak excretory time. In group FUM*po* there was already a fair degree of metabolism from parent toxin to the partially (and even fully) hydrolyzed forms, most pronounced for FB1 (61.4% FB1), followed by FB3 (43.4% FB3) and only minor metabolism was evident for FB2 (21.7% FB2). After esterase application (group FumD*po*), there was a general shift from parent toxin and the partially hydrolyzed forms for all three fumonisins FB1, FB2 and FB3 towards the fully hydrolyzed metabolites HFB. However, the extent of this metabolism differed somewhat between the three fumonisins: for FB1 (Figure 5a) there was a clear shift towards HFB1 in group FumD*po* compared to FUM*po*, comprising about 79.8% of the metabolite profile. This was also observed for FB3, albeit to a lesser degree, with about 64.5% HFB3 for FumD*po* (Figure 5c). Again, FB2 appeared to be the least hydrolysable toxin after esterase application (Figure 5b). Significant differences after 48 h in feces (Mann-Whitney-U-test) between the two oral groups with or without FumD application were found as follows (Figure 5): Significantly more FB1 was found in FUM*po* than in FumD*po* group (*p* = 0.013), whereas after FumD application significantly more HFB1 was detected (*p* = 0.005). Both pHFB1a and pHFB1b were significantly reduced in FumD*po* group (*p* = 0.030 for both). FB2 was also reduced significantly in FumD*po* group (*p* = 0.020) and HFB1 significantly increased (*p* = 0.003), whereas pHFB2a, pHFB2b showed no statistical difference. A significant reduction was also revealed in FumD*po* group for FB3 (*p* = 0.020) and pHFB3a (*p* = 0.045) but not for pHFB3b and HFB3.

## 3. Discussion

The aim of the present study was to investigate the toxicokinetics and bioavailability of fumonisins and its metabolites as well as the possible impact of an additive with fumonisin esterase activity. The study design was based on examining kinetics in blood, urine and feces after administering a fumonisins orally and intravenously as a single bolus, whereby the *iv* route served as a reference for the estimation of oral bioavailability. 

Serum concentrations of FB1 after *iv*-bolus suggested a 2-compartment model, resulting in a short distribution half-life t_1/2α_ of 0.08 h (6 min) followed by a longer elimination half-life t_1/2β_ of 0.57 h (±0.4 h; =36 ± 20 min, Table 3). In contrast, following *iv*-dosing of growing pigs with radioactively labeled FB1 (0.25 µCi [0.4 mg]/kg BW), Prelusky, Trenholm and Savard [26] showed that the level of radioactivity declined both in a tri-exponential and bi-exponential manner, depending on the individual pig, suggesting 3 or 2 compartments, respectively. Among others, the identification of additional compartments also depends on the blood sampling frequency which was different between this study and our own experiment. They detected an average half-life of the initial α-phase of less than 3 min (range 0.9–3.6 min) and a slower distribution β-phase t_1/2_ of 10.5 min (range 6.0–19.0 min). The following elimination γ-phase t_1/2_ of 182.6 min (range 142.6–224.3 min) was much longer than the 34 min observed in our experiment. Moreover, the mentioned authors used ^14^C-labelled FB1 and so measuring radioactivity as such and not the toxin itself [26]. Thus, total recovery might be higher since all breakdown compounds should be recovered but it is not possible to distinguish between the parent toxin and its break-down products. In our present experiment, we used the currently available analytical methods for parent toxin and its metabolites but we might be omitting metabolites yet unknown with no available analytical method at this time. In addition, compared to Prelusky, Trenholm and Savard [26], we also used a much lower dose of 100 µg FB1/kg BW whereas they utilized 400 µg/kg BW. For FB1 plus metabolites, we determined the same half-lives of distribution and elimination as for FB1 alone. This shows that there is no distinct difference in kinetics between FB1 and its metabolites (Table 1). 

For the total serum clearance Cl in the present study, we found 36 mL/kg BW/min (=6.7 L/kg/h), while Prelusky, Trenholm and Savard [26] reported a total plasma clearance of 9.14 ± 1.07 mL/min/kg BW. This could be partly due to using either plasma or serum, or more likely, due to the differing half-lives of distribution and elimination. In the present study, half-lives of elimination are shorter than in the study mentioned above [26] corresponding to a higher clearance in the present experiment (Table 1). In another study from Martinez-Larranaga et al. [27], administering 2 mg FB/kg BW *iv* to male Wistar rats and using a 2-compartment model for kinetic evaluation, the distribution half-life t_1/2α_ was comparable to the results of our study (0.15 h vs. 0.08 h) while elimination half-life t_1/2_β was much longer (1.03 h vs 0.57 h). Thus, the longer elimination half-life in rats might indicate a species-related difference in fumonisin toxicokinetics. 

The urinary total FB1 excretion amounted to ~8% of the provided *iv* bolus 6 h after exposure, representing peak excretion. FB1 alone represented 91% of the sum of FB1 plus metabolites in urine, whereas in serum the corresponding AUC ratio revealed a FB1 proportion of 93% suggesting a poor systemic FB1 metabolism. Prelusky, Trenholm and Savard [26] also found a high rate of urinary recovery of fumonisin-related radioactivity within the first 3 h after the bolus. While the maximum fractional FB1 excretion was reached after 6 h in urine, the respective maximum in feces was demonstrated after 48 h for all metabolites, suggesting biliary excretion and transit time of toxin residues distal of the *Ductus choledochus*. The assumption of substantial biliary FB1 excretion is also supported by the results of the study mentioned above [26] where the authors found that 71% of *iv* administered fumonisin could be recovered with bile, while 58% reached the feces suggesting reabsorption and urinary excretion and/or large intestinal fermentation. In the present experiment, the cumulative fecal excretion of FB1+m amounted to ~72% of that in urine, supporting the view of bile as a main excretory route for elimination. Moreover, the proportion of FB1 of the sum of FB1 and metabolites in feces amounted to 29% which is equivalent to a substantial intestinal metabolism of 71%. 

The distribution half-life t_1/2α_ of *iv* administered HFB1 was even shorter than for FB1*iv* but the elimination half-life was longer. V_d_ was 6 times higher than for FB1*iv* but varied considerably (1.2–22.4 L/kg BW). Cl was also four times higher in the HFB1*iv* compared to FB1*iv* group (6.7 and 1.8 L/kg/h respectively) which together with the differences in the volumes of distribution indicates that HFB1 is more rapidly cleared from the systemic circulation but also distributed more extensively into the extravasal space than FB1. HFB1 accounts for more than 96% of HFB1 plus metabolites after *iv* HFB1 administration in systemic circulation while only 93% of administered FB1 was recovered as parent toxin, which shows that FB1 is more intensively metabolized. 

Only a few studies on toxicokinetics of orally administered fumonisins in pigs were reported so far. In our trial FB1−m peaked at 9.5 h (t_max_), whereas FB1+m showed its peak somewhat later (t_max_ = 13.6 h). In contrast, other studies employing single bolus applications, showed much earlier peak concentrations in the blood stream, between 60–90 min [26] and 180 min [13]. This difference applied also for toxin recovery in other matrices, for example, 76.5% of initial FB1 was recovered after 84 h in feces, with peak excretion already in the first 24 h after gavage [13], contrasting to the 48 h in our trial. In both referred experiments fumonisins were applied on fasted pigs using a gavage technique, whereas pigs in our experiment consumed the FB1-dressed diet voluntarily within ~10 min. This distinction between fasted and fed status during oral toxin exposure appears to play a crucial role as in the fasted piglets absorption and fecal excretion of the parental FB1 was reported as much faster compared to our animals being exposed via feed. Moreover, the percentage of excreted parental FB1 in feces [13] was nearly tripled compared to our data (25% after 120 h) and rather equaled that of the sum of FB1 and its metabolites (73% FB1+m). Thus, the question is whether only little FB1 metabolism takes place in the intestinal tract of fasted piglets, which is perhaps due to an altered peristalsis compared to fed animals. In two studies, Fodor and colleagues investigated the excretory behavior of fumonisins in pigs fed continuously with fumonisin-dressed diets. In the first trial [28], they provided about half of our own FB1-dosis (50 mg/pig·d) continuously for 22 days and determined its fecal excretion at 13% of intake for a 5d-collection period during days 13 to 17 of exposure, albeit without determination of the metabolites. The second trial [24] determined the excretion during the first ten days of exposure and revealing higher daily fecal recoveries (72%). Additionally, the partially hydrolyzed fumonisins were also reported at 59% of the total fecal recovery. These data agree with our own findings, even taking the differences in mode of application (bolus via continuous exposure) into consideration. Thus, the absorption from and hydrolytic break-down in the intestinal lumen appears to be strongly influenced by the feeding status of the exposed animal.

The substantial higher V_d_ of FB1 alone (FB1−m) after *po* FB1 administration (8.7 L·kg·BW^−1^) compared to the *iv* route (1.8 L·kg·BW^−1^) might be explained by the marked FB1 metabolism putatively occurring in the digestive tract of *po* dosed pigs. This resulted in lower blood levels of FB1 compared to the *iv* route of administration. On the other hand, when metabolites were included in the kinetic evaluation after *po* FB1 administration, the V_d_ was slightly lower compared to FB1 data alone (7.7 L·kg·BW^−1^). This hints at differences in the distribution pattern between FB1 and its metabolites consisting of pHFB1a, pHFB1b and HFB1. Interestingly, the V_d_ of HFB1 was substantially higher (11.0 L·kg·BW^−1^) than for FB1 when the toxins were administered via the *iv* (1.8 L·kg·BW^−1^) route. As *iv* kinetics of both toxins was only slightly influenced by metabolites, the higher V_d_ of HFB1 might point a more pronounced extravasal distribution. Based on the findings by Prelusky (1994) that suggest intensive biliary excretion and entero-hepatic cycling of FB1 it seems reasonable to assume that HFB1 is even more efficiently biliary excreted and/or entero-hepatically recycled than FB1. The predicted octanol-water partitioning coefficients from structures (k_ow_, expressed as logarithmic ratio log*P*) of FB1 and HFB1 amount to 2.20 and 1.25 (ChemSpider, Royal Society of Chemistry 2015), respectively, suggesting FB1 to be more lipophilic than HFB1. Thus, a more pronounced distribution of HFB1 into adipose tissues seems less probable than the biliary recycling. The more pronounced entero-hepatic cycling of HFB1 is further supported by the delayed absorption half-life (t_1/2α_) and peak concentration of the sum of FB1 and metabolites (including HFB1) which, in turn, could be related to the less pronounced lipophilicity of HFB1 hampering simple diffusion of the double-lipid layer of the enterocytes.

The low oral bioavailability of 3.1% for FB1 agrees with those of 4.1% as reported by Prelusky, Trenholm, Rotter, Miller, Savard, Yeung and Scott [25] for pigs and for rats of 3.5% [27]. Bouhet and Oswald [29] discussed whether the poor absorption and consequently the low bioavailability of fumonisins are due to a poor transport across the epithelium of the intestine or due to strong association of fumonisin with the intestinal content. Furthermore, the so called “fumonisin paradox” has been proposed by Shier [14] which describes the apparent contradiction between the potent toxicity of fumonisins in various species and the poor absorption. Besides single bolus experiments, the absorption can also be determined experimentally in animals exposed to the toxin for a certain period of time to reach a steady state where a balance between intake and excretion is enabled. Using this technique [30], an apparent absorption rate of approximately 4% up to the end of the ileum was determined in 8-weeks old T-cannulated pigs fed a fumonisin-contaminated diet for 10 days (45 mg FB1, 8.6 mg FB2, 4.6 mg FB3/kg feed). In a similar experimental setting, the absorption rate of 3.9% was reported by Fodor, Balogh, Weber, Miklos, Kametler, Posa, Mamet, Bauer, Horn, Kovacs and Kovacs [24] in T-cannulated pigs, confirming the low fumonisin bioavailability. The detection of fumonisin residues in intestinal cells of a non-human primate after *po* fumonisin administration (25% of the dose) suggests that the toxin enters this cell type [31] but the mechanism of fumonisin absorption by enterocytes are still largely unknown. Although we cannot exclude that a certain percentage of the administered *po* dose is stored in the body, the low cumulative urinary recovery of fumonisin residues supports the low systemic bioavailability of fumonisins after *po* administration as well. Only 0.24% (FB1−m) and 0.56% (FB1+m) of the dose were recovered with urine. These results agree with other findings reporting urine recovery at 0.93% and 0.59% of the FB1 bolus administered *po* [13,26]. The high fecal recoveries of FB1−m and of FB1+m of 25% and 72%, respectively, are an additional indication both for the poor toxin absorption and the low systemic bioavailability. The large difference between fecal excretion of FB1 alone and FB1 plus metabolites hints at extensive FB1 metabolism in the digestive tract. The porcine intestinal microbiota appears to form the partially hydrolyzed forms of fumonisin B1 preferentially and this applies also for fumonisin B2 and B3 in a similar manner, albeit to a lesser extent dependent on the fumonisin type. Our data also indicate that there seems to be no preferred degradation into form a or b of pHFB as both types were verified in fecal material to a comparable extent. In vitro data indicate the ability of the porcine cecal microbiota to effectively hydrolyze fumonisin [32] to its partially hydrolyzed form. Even *in vivo* conversion of FB1 into pHFB1 at ~4% was reported in piglets fed with fumonisin-dressed feed [24]. However, so far there is no indication of which part of the microbiota could possess such esterase activities and exercise them upon fumonisin exposure.

The metabolism of fumonisin into the respective break-down products was distinctly different from the degradation discussed above when fumonisin esterase (FumD) was applied as a feed additive concomitantly with fumonisin via the morning feed. Excretion of parent toxin was dramatically reduced by more than 90% in urine and feces and a marked shift from the partially to the fully hydrolyzed products was determined. This was particularly apparent at fecal level for all three fumonisin types, albeit to a slightly lesser extent for FB2 and FB3. This impact was also reflected in a significant reduction of bioavailability upon use of fumonisin esterase, for FB1 alone by 90% and FB1 including metabolites by 85%. Our results support former studies with pigs where the efficacy of the enzyme was proven indirectly by influencing the well-established biomarker for fumonisin exposure, that is, the sphinganine/sphingosine ratio [22]. 

In conclusion, *iv* serum toxicokinetics of FB1 suggested a 2-compartment model and showed a 6 min distribution half-life followed by a 36 min elimination half-life. A four-times higher Cl together with a six-times higher V_d_ compared to FB1*iv* group indicates that after *iv* application, HFB1 is more rapidly cleared from systemic circulation but distributed more extensively into the extravasal space than FB1. Furthermore, our toxicokinetic measurements confirmed the low bioavailability of fumonisin *in vivo* (3% FB1) and feces as main excretory route after oral exposure. We could also report substantial fumonisin degradation into their partially hydrolyzed forms in the gastro-intestinal tract, most likely executed by the resident microbiota. The actual mechanism behind this degradation remains to be elucidated.

Moreover, the efficacy of a fumonisin esterase as feed additive could clearly be demonstrated by a 90% reduction in FB1-bioavailability (0.3%) and more than 90% reduced excretion of FB1, FB2 and FB3 via urine and feces. At a fecal level, the esterase application resulted in a dramatic shift in metabolite pattern from the detrimental FB1 to the reportedly less toxic, fully hydrolyzed HFB1. This change was also observed for FB2 and FB3, albeit for FB2 the degradation into the fully hydrolyzed form was detected to a lower extent.

## 4. Materials and Methods

The experiment was conducted according to the European Community regulations concerning the protection of experimental animals and the guidelines of the German Animal Welfare Act and was approved by the Lower Saxony State Office for Consumer Protection and Food Safety (file number 33.92-42502-04-13/1153, date of approval: 11.07.2013).

### 4.1. Animals, Housing and Diet

The study was performed with a total of 31 barrows (German Landrace, Mariensee, Germany), housed individually and fed restrictively a barley-based basal diet (2 × 700 g/d), formulated to meet or exceed requirements according to GfE recommendations (Table S1 [33]), for the total experimental period. Diet was analyzed by HPLC-MS/MS (Romer Labs Diagnostic GmbH, Tulln, Austria) and values were all below level of detection (LOD) for FB1, FB2, FB3 (LOD = 20 μg/kg) and aflatoxin B1, B2, G1, G2 (LOD = 0.2 μg/kg). Pigs (aged 10 weeks at the start of experiment) were housed in floor pens for 21 days and thereafter moved to metabolism crates enabling quantitative collection of feces and urine in the subsequent experimental period. After a four-day adaption period in metabolism crates and fasting overnight, body weight (BW) of pigs was measured (34.4 kg ± 2.7 kg BW) and surgical implantation of indwelling venous catheters (Silastic^®^, Medical Grade Tubing. 1.57 mm ID × 3.18 mm OD, Dow Corning, Midland, MI, USA) in both *Venae jugulares externae* was performed under sterile conditions on day 25 as described by Tesch et al. [34]. After surgery, the animals recovered for one day before the actual toxicokinetic study was started on day 27. Catheters were flushed regularly with heparinized saline solution (2 mL heparin/ 500 mL physiological saline) to ensure patency.

### 4.2. Toxicokinetic Study

For the kinetic study, pigs received one of five treatments on day 27 (Table 6): [1] CON (basal diet, 0.9% NaCl *iv*), [2] FB1*iv* (basal diet, 100 µg FB1/kg BW as single bolus), [3] HFB1*iv* (basal diet, 56.2 µg HFB1/kg BW; single bolus), [4] FUM*po* (3377 nmol FB1 + 1367 nmol FB2 + 584 nmol FB3/kg BW, 0.9% NaCl *iv*), [5] FumD*po* (3321 nmol FB1 + 1344 nmol FB2 + 575 nmol FB3/kg BW and 240 U FumD/kg feed, 0.9% NaCl *iv*).

FB1 and HFB1 standards for both *iv* treatments were diluted with physiological saline adjusting necessary concentrations whereby FB1 was obtained from Romer Labs GmbH (Tulln, Austria; 97.6% pure). HFB1 was produced from the same FB1 standard by stoichiometric transformation with KOH as previously described (>98% pure [23]). From lyophilized standards, stock solution was prepared with sterile 0.9% NaCl whereby concentration of stock solution was supposed to be 600 µg FB1/mL and 331.7 µg HFB1/mL, respectively. The stock solution was then used as basis to calculate injection volume (FB1: 100 µg FB1/kg BW; HFB1: 56.2 µg HFB1/kg BW), which was 5.6 ± 0.3 mL per pig for *iv* treated animals. After application, catheters were flushed with physiological saline to attain a total injection volume of 25 mL and the other groups (FUM*po*, FumD*po* and CON group) received 25 mL of physiological saline. For both oral treatments, FUM*po* and FumD*po*, fumonisin culture material, derived from *Fusarium verticillioides* (10.47 mg FB1/g, 4.18 mg FB2/g and 1.24 mg FB3/g culture material, Romer Labs GmbH, Tulln, Austria) as previously described by Grenier et al. [35], was used on top of the morning ration (basal diet) and a fumonisin esterase preparation (FUM*zyme*^®^, BIOMIN, Tulln, Austria) for FumD*po* treatment. These ingredients were blended with the morning basal feed using a 1 kg ploughshare mixer (Gebr. Lödige, Paderborn, Germany) for 4 min on day 26 and a sample of each ration was taken and analyzed by HPLC-MS for fumonisin concentration (Romer Labs GmbH, Tulln, Austria) and revealed 80.9 ± 6.9 mg FB1, 33.2 ± 3 mg FB2 and 6.1 ± 1.9 mg FB3/kg feed in both contaminated rations of group FUM*po* and FumD*po*.

Blood samples were collected directly before treatment and then 5, 10, 15, 20, 30, 45 min, 1, 1.5, 2, 2.5, 3, 3.5, 4, 6, 12, 24, 48, 72, 96 and 120 h after treatment (Serum Monovette, Sarstedt AG & Co., Sarstedt, Germany). Moreover, urine and feces were collected quantitatively for 12 h prior to toxin application and then at 6, 12, 24, 48, 72, 96 and 120 h *post* treatment in containers placed under the metabolism crates. After urine volume and feces weight were recorded for balance calculations, a representative sample was taken from each gross sample and stored at −20 °C until analysis. Feces samples were blended, freeze-dried and ground (La Moulinette, Moulinex) before being stored at −20 °C for further analysis.

Clinical examination (respiratory parameters, cardiovascular system and heart rate, consciousness, behavior, skin and bristles, central nervous system, gastrointestinal tract and body temperature) of pigs was performed once before the experiment started and subsequently prior to morning feeding once every day to monitor animal health. However, detailed clinical data are not part of this paper and are therefore not presented here.

Animals were sacrificed by exsanguination following electrical stunning 120 h post treatment (day 31) and tissue (muscle, fat, kidney, liver, lungs, spleen; data not shown) and fluid samples (bile, *liquor cerebrospinalis*) were collected for analysis.

### 4.3. Mycotoxin Analysis

Mycotoxin standards of FB1, FB2 and FB3 were purchased from Romer Labs GmbH (Tulln, Austria). Partially and fully hydrolyzed forms of all three toxins were produced from these standards by enzymatic hydrolysis, using fumonisin esterase, as described for FB1 by Schwartz-Zimmermann et al. (submitted). Similarly, fully ^13^C-labelled FB1 (Romer Labs GmbH, Tulln, Austria) was subjected to enzymatic hydrolysis. The resulting mixture of FB1 and its partial and full hydrolysis products was added to serum and urine samples as internal standard.

Blood samples were kept upright at room temperature for at least 30 min for clotting, subsequently centrifuged at 2123 *g* for 15 min and serum aliquots were stored at −20 °C until analysis. After thawing, a volume of 300 µL serum was mixed with ^13^C-labelled internal standard (a mixture of FB1 and its partial and full hydrolysis products) and 900 µL of methanol/acetonitrile (50/50, *v*/*v*) were added. Samples were shaken at room temperature for 30 min and centrifuged (2800 *g*). The pellets were re-extracted twice with 200 µL of acetonitrile/water/formic acid (50/49/1, *v*/*v*/*v*). The combined supernatants were dried under vacuum, re-dissolved in 300 µL of acetonitrile/water/formic acid (50/49/1, *v*/*v*/*v*) and cleared by centrifugation prior to analysis.

Creatinine content of urine was determined by HPLC-MS/MS [36] in order to account for varying water content of urine samples. For mycotoxin analysis, 150 µL aliquots of urine (diluted with water to 4 mM creatinine content) were mixed with 10 µL of ^13^C-labelled internal standard. After addition of 290 µL methanol/formic acid (99/1, *v*/*v*) the samples were vortexed, stored for 15 min at −20 °C, shaken for 10 min at room temperature and cleared by centrifugation before analysis as described for serum. 

FB1, 2 and 3, their partially and completely hydrolyzed metabolites were analyzed in lyophilized feces as previously described [22].

Details to mean recoveries, limit of detection and quantification are provided in Table 7 for each analyzed matrix. 

### 4.4. Calculations and Statistics

All statistical analyses were carried out using Dell Statistica version 13 software for the Windows™ operating system (Dell Inc., Tulsa, OK, USA) and SAS 9.4 (SAS Institute Inc., Cary, NC, USA) for post-hoc test significances. Values < LOD were determined as 0.

#### 4.4.1. Serum Kinetics of the *iv* Groups

FB1 and metabolite serum concentrations (HFB1, pHFB1a, pHFB1b) of the *iv* dosed pigs were characterized by a two-compartment model and could be described by a common pharmacokinetic bi-exponential regression of the form [37]:

(1) $$c\left(t\right)={A*e}^{-\alpha *t}+{B*e}^{-\beta *t},$$

where c is the fumonisin concentration at time t (h), A and B the initial concentration at t = 0 and α and β are the rate constants of the distribution and elimination phase, respectively. The non-linear curve fitting module of the Statistica version 13 was used to fit data to Equation (1). The half-life was calculated as t12α=ln(2)α for distribution phase and t12β=ln(2)β for elimination. The area under the serum-time curve AUC for FB1 was defined by $AUC=\frac{A}{\alpha }+\frac{B}{\beta }$, whereas the plasma clearance Cl of FB1 was determined by $Cl=\frac{D}{AUC}$, where D is the dosage. The apparent volume of distribution was estimated by Vd=Cl∗t12βln(2). 

#### 4.4.2. Serum Kinetics of the Oral Groups 

Serum concentrations of FB1 and its metabolites after *po* FUM exposure were fitted according to the Bateman function [38]:

(2) $$c\left(t\right)=\frac{F*D}{V_{d}}*\frac{k_{a}}{k_{e}-k_{a}}*(e^{-k_{e}*t}-e^{-k_{a}*t}),$$

where c(t) = fumonisin and metabolite concentration (ng/mL) at time t (h), F is the fraction of the substance appearing in the systemic circulation of the FB1, D = dose of FB1 (nmol·kg·BW^−1^) administered *po*, V_d_ = apparent volume of distribution (L/kg), k_a_ is the first order rate constant of invasion (1/h), k_e_ is the first order rate constant of elimination (1/h) and t = time (h). Only four out of six pigs followed the typical shape of the Bateman function and were used for data evaluation (*n* = 4). In contrast to group FUM*po*, the serum concentrations of group FumD*po* were much lower and did generally not follow the Bateman function. Therefore, a kinetic evaluation for this group was not feasible. 

Based on the fitted regression coefficients following further kinetic parameters were derived: half-lives of absorption and elimination as t12a=ln(2)ka (h) and t12e=ln(2)ke (h), respectively; whole body clearance as Cl=ln(2)∗Vt12e (L∙kg^−1^∙h^−1^); the area under the plasma substance concentration x time curve as $AUC=\frac{\frac{F*D}{V}}{k_{e}}$ (µg∙L^−1^∙h) and the mean residence time MRT (h) of the substance in the invaded compartment as the quotient between the first moment AUC (AUMC, µg∙L^−1^∙h∙h) and the zero moment AUC (AUC, µg∙L^−1^∙h); the time at maximum substance plasma concentration was estimated as $t_{max}=\frac{1}{\left(k_{a}-k_{e}\right)*ln\left(k_{a}-k_{e}\right)}$ (h) and the corresponding maximum plasma concentration C_max_ by solving of Equation (2) for t = t_max_ (µg∙L^−1^).

#### 4.4.3. Bioavailability 

The systemic bioavailability (F_AUC_) of FB1 alone or with consideration of the metabolites was estimated for groups FUM*po* and FumD*po* as follows (Equation (3)):

(3) $$F_{\#}=\frac{AUC_{po\#}*D_{iv}}{D_{po\#}*AUC_{ivMean}}*100,$$

where “**#**” represents values of individual animals. Since kinetics after *iv* and *po* toxin application was not determined with the same animals the mean value for the AUC*_iv_* (Section 2.1.4, Table 1 and Table 2) needed to be used for bioavailability determination (AUC*_iv_*_Mean_). AUC*_po_* were either estimated as described in Section 2.1.4 or simply determined geometrically by using the trapezoidal rule. The latter was applied particularly for group FumD*po* which could not be evaluated kinetically (see Section 2.3).

The relative bioavailability (F_rel_) after FumD*po* administration was estimated by (Equation (4)):

(4) $$F_{rel}=\frac{AUC_{FumDpo}*D_{FUMs\;po}}{D_{FumDpo}*AUC_{FUMspo}}*100,$$

where AUC_FumD*po*_ and AUC_FUM*po*_ are the means calculated from the individual AUCs for FumD*po* and FUM*po* group (*n* = 6), respectively. D_FumD*po*_ and D_FUM*po*_ are the corresponding average oral doses.

AUC and F for FB1 alone were statistically evaluated via Mann-Whitney-U-test for non-parametric data, whereas AUC and F for FB1 plus metabolites were evaluated via Students *t*-test for independent data sorted in groups. 

#### 4.4.4. Urine and Feces Balance Calculations

The recovery of fumonisin B1, B2 and B3 and their metabolites in urine and feces was calculated as percentage of the administered parent toxins, based on the quantitative collection of the two matrices (12 h prior to toxin application and then at 6, 12, 24, 48, 72, 96 and 120 h *post* treatment).

The cumulative excretion of urine and feces was estimated by (Equation (5)):

(5) $$cum.\;excretion\;\left(\%\;of\;intake\right)=\Sigma_{t=0}^{t_{i}}\frac{toxin\;excreted\;\left[ng\right]}{toxin\;administered\;\left[ng\right]}*100,$$

whereby this refers to the totally administered toxin (*iv* or *po*) per subject and its total excretions measured in the respective matrices (feces: g; urine: mL) up to the sampling point t_i_ (h).

Statistics were evaluated with PROC MIXED in SAS 9.4 (SAS Institute Inc., Cary, NC, USA) with group and time as main factors and time as repeated measure. Post hoc significances were determined via Student’s-*t*-test for normally distributed data (LSmeans ± SD) and Mann-Whitney-U test for non-Gaussian distributed parameters (Median/Min-Max). 

## Figures and Tables

**Table 1:** Summary of the serum toxicokinetics of FB1 administered *iv* (FB1*iv*) as a single bolus of 139 nmol·kg·BW^−1^ in barrows (*n* = 6).

**FB1−m**								
**Animal**	4	5	15	18	23	29	Mean	SD
**BW (kg)**	31.0	33.0	35.0	35.4	32.3	35.0	33.6	1.8
**FB1 (nmol·kg·BW^−1^)**	139	139	139	139	139	139	139	0
**A (nmol·L^−1^)**	410.0	200.3	238.7	766.9	1022.9	573.7	535.4	319.0
**α (h^−1^)**	14.9	5.6	7.4	16.4	12.2	8.1	10.8	4.4
**B (nmol·L^−1^)**	94.8	7.4	10.3	194.0	440.7	119.9	144.5	161.3
**β (h^−1^)**	2.8	0.6	0.8	2.2	2.4	1.5	1.7	0.9
**t_1/2α_ (h)**	0.05	0.12	0.09	0.04	0.06	0.09	0.08	0.03
**t_1/2β_ (h)**	0.25	1.26	0.83	0.32	0.29	0.47	0.57	0.40
**V_d_ (L·kg·BW^−1^)**	0.8	5.1	3.7	0.5	0.2	0.6	1.8	2.1
**Cl (L·kg·BW^−1^·h^−1^)**	2.3	2.8	3.1	1.0	0.5	0.9	1.8	1.1
**AUC (nmol·L^−1^·h)**	61	49	45	135	266	152	118	86
**RSD (nmol·L^−1^)**	1.9	1.4	1.7	3.1	3.6	5.8	2.9	1.7
***r*^2^**	0.998	0.999	0.997	0.998	0.999	0.998	0.998	0.001
**FB1+m**								
**A (nmol·L^−1^)**	483.1	214.9	260.4	843.1	1122.1	636.0	593	349
**α (h^−1^)**	16.0	6.0	7.8	15.9	12.1	8.1	11.0	4.3
**B (nmol·L^−1^)**	98.1	8.7	10.6	205.9	481.1	135.7	156.7	175.9
**β (h^−1^)**	2.9	0.6	0.7	2.1	2.4	1.5	1.7	0.9
**t_1/2α_ (h)**	0.04	0.12	0.09	0.04	0.06	0.09	0.07	0.03
**t_1/2β_ (h)**	0.24	1.22	0.94	0.33	0.29	0.46	0.58	0.40
**V_d_ (L·kg·BW^−1^)**	0.8	4.8	3.9	0.4	0.2	0.6	1.8	2.0
**Cl (L·kg·BW^−1^·h^−1^)**	2.2	2.7	2.9	0.9	0.5	0.8	1.7	1.1
**AUC (nmol·L^−1^·h)**	64	51	48	150	293	169	129	96
**AUC_FB1−m%_ (%)**	95	96	93	90	91	90	93	3
**RSD (nMol·L^−1^)**	2.0	1.4	1.8	3.6	4.3	6.0	3.2	1.8
***r*^2^**	0.998	0.998	0.997	0.999	0.999	0.997	0.998	0.001

BW = body weight; −m = FB1 alone; +m = FB1 plus metabolites: HFB1, pHFB1a, pHFB1b; *A, B* = initial concentrations for distribution and elimination, respectively, at *t* = 0; *α* and *β* = rate constants of distribution or elimination phase; t_1/2α_ and t_1/2β_ = half-lives of distribution and elimination, respectively; V_d_ = apparent volume of distribution, Cl = serum clearance, AUC = area under the time vs. concentration curve; AUC_FB1−m%_ = AUC of FB1 alone without metabolites compared to FB1 plus sum of metabolites in %; RSD = residual standard deviation of the regression; *r*^2^ = coefficient of determination; SD = standard deviation. Data were evaluated with Students *t*-test for independent data sorted in groups (for parametric data).

**Table 2:** Summary of the serum toxicokinetics of HFB1 administered *iv* (HFB1*iv*) in a single bolus of 139 nmol·kg·BW^−1^ in barrows (*n* = 5).

**HFB1−m**							
**Animal**	1	8	13	21	28	Mean	SD
**BW (kg)**	34.9	35.6	33.5	34.0	32.0	34.0	1.4
**HFB1 (nmol·kg·BW^−1^)**	139	139	139	139	139	139	0
**A (nmol·L^−1^)**	69.5	297.3	205.2	122.8	724.1	283.8	260.8
**α (h^−1^)**	10.8	20.5	14.6	10.7	15.9	14.5	4.1
**B (nmol·L^−1^)**	8.8	1.0	1.2	42.2	48.6	20	23
**β (h^−1^)**	1.2	0.4	0.4	2.9	1.5	1.3	1.0
**t_1/2α_ (h)**	0.06	0.03	0.05	0.07	0.04	0.05	0.02
**t_1/2β_ (h)**	0.58	1.82	1.92	0.24	0.47	1.01	0.80
**V_d_ (L·kg·BW^−1^)**	8.5	21.3	22.4	1.8	1.2	11.0	10.3
**Cl (L·kg·BW^−1^·h^−1^)**	10.1	8.1	8.1	5.4	1.8	6.7	3.2
**AUC (nmol·L^−1^·h)**	14	17	17	26	78	30	27
**RSD (nmol·L^−1^)**	0.1	0.7	1.6	2.8	1.7	1.4	1.0
***r*^2^**	0.999	0.998	0.987	0.983	0.999	0.993	0.008
**HFB1+m**							
**A (nmol·L^−1^)**	69.8	299.1	205.8	138.8	724.5	287.6	258.5
**α (h^−1^)**	11.2	20.6	14.7	7.4	15.6	13.9	4.9
**B (nmol·L^−1^)**	9.6	1.1	1.2	8.9	51.3	14.4	21.0
**β (h^−1^)**	1.2	0.4	0.3	0.9	1.5	0.9	0.5
**t_1/2α_ (h)**	0.06	0.03	0.05	0.09	0.05	0.06	0.02
**t_1/2β_ (h)**	0.56	1.81	2.37	0.80	0.45	1.20	0.85
**V_d_ (L·kg·BW^−1^)**	8.0	21.0	26.2	5.5	1.1	12.4	10.7
**Cl (L·kg·BW^−1^·h^−1^)**	9.9	8.0	7.7	4.8	1.7	6.4	3.2
**AUC (nmol·L^−1^·h)**	14	17	18	29	80	32	28
**AUC_HFB1−m%_ (%)**	98.6	98.9	95.3	89.6	98.1	96.1	3.9
**RSD (nmol·L^−1^)**	0.1	0.8	1.6	3.0	2.0	1.5	1.1
***r*^2^**	0.999	0.995	0.987	0.981	0.998	0.992	0.008

BW = body weight; −m = HFB1 alone; +m = HFB1 plus metabolites: pHFB1a, pHFB1b; *A*, *B* = initial concentrations for distribution and elimination, respectively, at *t* = 0; *α* and *β* = rate constants of distribution or elimination phase; t_1/2α_ and t_1/2β_ = half-lives of distribution and elimination, respectively; V_d_ = apparent volume of distribution, Cl = serum clearance, AUC = area under the time vs. concentration curve; AUC_HFB1−m%_ = AUC of HFB1 alone without metabolites compared to HFB1 plus sum of metabolites in %; RSD = residual standard deviation of the regression; *r*^2^ = coefficient of determination; SD = standard deviation. Data were evaluated with Students *t*-test for independent data sorted in groups (for parametric data).

**Table 3:** Summary of the serum toxicokinetics of FB1 administered orally (FUM*po*) at 3377 nmol·kg·BW^−1^ (FB1) + 1367 nmol·kg·BW^−1^ (FB2) + 584 nmol·kg·BW^−1^ (FB3) in barrows (*n* = 4).

**FB1−m**							
**Animal**		2	14	17	25	Mean	SD
**BW (kg)**		31.6	37.5	35.5	32.0	34.2	2.8
**FB1 (nmol·kg·BW^−1^)**		3683	3103	3278	3637	3425	281
**k_a_ (h^−1^)**		0.15	2.22	0.25	0.08	0.68	1.03
**k_e_ (h^−1^)**		0.03	0.02	0.04	0.08	0.04	0.03
**C_0_ (nmol·L^−1^)**		3.6	1.3	3.3	5.5	3.4	1.7
**c_max_ (nmol·L^−1^)**		2.5	1.3	2.4	2.0	2.1	0.5
**t_max_ (h)**		13.9	2.2	8.7	13.1	9.5	5.4
**t_1/2ka_ (h)**		4.6	0.3	2.7	9.1	4.2	3.7
**t_1/2ke_ (h)**		26.0	38.5	17.7	9.1	22.8	12.5
**V_d_ (L·kg·BW^−1^)**		7.3	10.1	6.5	10.7	8.7	2.1
**Cl (L·kg·BW^−1^·h^−1^)**		0.2	0.2	0.3	0.8	0.4	0.3
**AUC (nmol·L^−1^·h)**		135	73	85	71	91	30
**F_AUC_ (%)**		4.3	2.8	3.1	2.3	3.1	0.8
**F (%)**		0.007	0.004	0.007	0.016	0.009	0.005
**MRT (h)**		33.6	28.4	21.2	18.5	25.4	6.9
**RSD (nmol·L^−1^)**		0.5	0.3	0.4	0.4	0.4	0.1
***r*^2^**		0.684	0.655	0.764	0.672	0.694	0.048
**FB1+m**							
**k_a_ (h^−1^)**	0.11		0.26	0.11	0.07	0.14	0.08
**k_e_ (h^−1^)**	0.03		0.02	0.04	0.07	0.04	0.02
**c_0_ (nmol·L^−1^)**	9.0		2.8	5.9	5.8	5.9	2.5
**c_max_ (nmol·L^−1^)**	5.7		2.3	3.2	2.1	3.3	1.7
**t_max_ (h)**	16.6		10.6	13.6	13.7	13.6	2.5
**t_1/2ka_ (h)**	6.2		2.7	6.1	9.5	6.1	2.8
**t_1/2ke_ (h)**	25.3		32.4	15.5	9.5	20.7	10.2
**V_d_ (L·kg·BW^−1^)**	1.7		7.4	10.3	11.3	7.7	4.3
**Cl (L·kg·BW^−1^·h^−1^)**	0.05		0.16	0.46	0.83	0.4	0.3
**AUC (nmol·L^−1^·h)**	328		132	132	80	168	109
**AUC_FUM-m%_ (%)**	41.3		55.0	64.4	89.5	62.6	20.3
**F_AUC_ (%)**	9.6		4.6	4.3	2.4	5.2	3.1
**F**	0.004		0.007	0.019	0.018	0.012	0.008
**MRT (h)**	34.7		31.5	25.0	23.2	28.6	5.4
**RSD (nmol·L^−1^)**	1.2		0.5	0.5	0.5	0.7	0.4
***r*^2^**	0.464		0.604	0.783	0.655	0.627	0.132

BW = body weight; −m = FB1 alone; +m = FB1 plus metabolites: HFB1, pHFB1a, pHFB1b; *k_a_* and *k_e_* = rate constants of absorption or elimination phase; c_0_ = initial concentration corresponding to elimination; c_max_ = maximum serum concentration, t_max_ = time corresponding to C_max_; t_1/2ka_ and t_1/2ke_ = half-lives of absorption and elimination, respectively; V_d_ = apparent volume of distribution, Cl = serum clearance, AUC = area under the time vs. concentration curve; F = fraction of the toxin appearing in the systemic circulation (%); F_AUC_ = AUC after *po* administration of the toxin divided by the mean AUC after *iv* administration; dose-corrected for the respective doses (see Table 4) and multiplied by 100; MRT = mean residence time of toxin in the invaded compartment; RSD = residual standard deviation; *r*^2^ = coefficient of determination; AUC_FUM-m%_ = AUC of FB1 alone without m compared to FB1 plus sum of metabolites in %; SD = standard deviation. Data were evaluated with Students *t*-test for independent data sorted in groups (for parametric data).

**Table 4:** Summary of the area under the curve (AUC) and bioavailability (F) of FB1 alone (FB1−m) and the sum of FB1 and metabolites (FB1+m) in serum of pigs administered a single bolus of 120 mg FB1 + 48 mg FB2 + 14 mg FB3/kg diet *po* with fumonisin esterase (FumD*po*, 240 U/kg diet, *n* = 6) or without (FUM*po*, *n* = 4).

	FB1−m			FB1+m		
	FUM*po*	FumD*po*	*p*-Value	FUM*po*	FumD*po*	*p*-Value
**AUC (nMol·L^−1^·h)**	91.0 (12)	9.0 (15.8)	<0.01	168.0 (45)	27.7 (20.0)	0.01
**F_AUC_ (%); absolute**	3.1 (0.4)	0.3 (0.6)	<0.01	5.2 (1.3)	0.8 (0.6)	0.01

AUC = area under the time vs. concentration curve, calculated using the trapezoid method (nmol·L^−1^·h); F_AUC_, absolute = AUC after *po* administration of the toxin divided by the mean AUC after *iv* administration; dose-corrected (see Table 1). Data are presented as means (±SD) and comparison between treatments was performed with a Student’s *t*-test.

**Table 5:** Cumulative urinary and fecal excretion of FB1 alone (FB1−m) and the sum of FB1 and its metabolites (FB1+m) after 120 h as percentage of exposure (median, *n* = 6/group).

	Urinary Excretion %		Fecal Excretion %	
	FB1−m	FB1+m	FB1−m	FB1+m
**FB1*iv***	10.56 ^a^ (8.33–13.44)	11.80 ^a^ (10.33–14.35)	1.88 ^a^ (0.00–3.76)	8.57 ^a^ (0.00–13.53)
**HFB1*iv***	<LOD	3.95 ^b^ (0.67–6.48)	0.00 ^a^ (0.00–1.71)	0.40 ^b^ (0.00–4.04)
**FUM*po***	0.24 ^c^ (0.12–0.56)	0.56 ^c^ (0.26–1.56)	25.05 ^b^ (8.51–55.51)	72.92 ^c^ (52.49–93.66)
**FumD*po***	0.012 ^b^ (0.001–0.061)	0.234 ^c^ (0.045–1.256)	1.34 ^a,c^ (0.22–2.26)	58.81 ^c^ (51.34–80.05)

−m = FB1 alone; +m = FB1 plus metabolites: HFB1, pHFB1a, pHFB1b; Data (median, min-max) with unlike superscripts in one column are significantly different from each other (Mann-Whitney-U-test, *p* < 0.05).

**Table 6:** Experimental design.

Group	Fumonisin Application	Dose (nmol·kg·BW^−1^)	Fumonisin Esterase (U/kg feed)	*n*
**CON**	-	-	-	6 (+1 ^‡^)
**FB1*iv***	100 µg FB1/kg BW *iv* bolus	139 FB1	-	6
**HFB1*iv***	56.2 µg HFB1/kg BW *iv* bolus	139 HFB1	-	6
**FUM*po***	culture material on top of morning ration, *po* bolus (calculated: 120 mg FB1 + 48 mg FB2 + 14 mg FB3/kg diet)	3377 FB1 ^†^ 1367 FB2 ^†^ 584 FB3 ^†^	-	6
**FumD*po***	culture material on top of morning ration, *po* bolus (calculated: 120 mg FB1 + 48 mg FB2 + 14 mg FB3/kg diet)	3321 FB1 ^†^ 1344 FB2 ^†^ 575 FB3 ^†^	240	6

^†^ Calculation: based on analysis of culture material (Romer Labs GmbH, Tulln, Austria) and animals’ body weight (BW) at time of application, ^‡^ One pig in CON removed its venous catheter and thus only urine and feces sampling was obtained for this individual.

**Table 7:** Mean recovery (%), limit of detection (LOD) and limit of quantification (LOQ) of fumonisins and their metabolites in different matrices.

		FB1	HFB1	pHFB1a	pHFB1b	FB2	HFB2	pHFB2a	pHFB2b	FB3	HFB3	pHFB3a	pHFB3b
	**Recovery (%)**	58	-	-	-	58	-	-	-	58	-	-	-
**Feed ^1^**	**LOD (ng/g)**	20	-	-	-	20	-	-	-	20	-	-	-
	**LOQ (ng/g)**	-	-	-	-		-	-	-		-	-	-
	**Recovery (%)**	99	105	94	93	91	-	-	-	95	-	-	-
**Serum**	**LOD (ng/mL)**	0.10	0.10	0.05	0.09	0.27	-	-	-	0.22	-	-	-
	**LOQ (ng/mL)**	0.30	0.30	0.15	0.27	0.80	-	-	-	0.66	-	-	-
	**Recovery (%)**	103	104	101	94	106	101	101	95	105	93	109	103
**Urine**	**LOD (ng/mL)**	0.90	1.4	0.27	0.36	0.90	0.27	0.18	0.27	0.18	0.36	0.18	0.18
	**LOQ (ng/mL)**	3.0	4.5	0.90	1.2	3.0	0.90	0.60	0.90	0.60	1.2	0.60	0.60
	**Recovery (%)**	105	104	101	101	105	103	98	99	110	102	97	106
**Feces**	**LOD (ng/g)**	35	131	25	33	185	217	16	10	121	82	10	10
	**LOQ (ng/g)**	116	437	83	108	618	725	52	21	405	274	23	32

^1^ analyzed by Romer Labs, Austria.

## Supplementary Materials

The following are available online at http://www.mdpi.com/2072-6651/10/4/150/s1. Table S1: Composition of basal diet.
