# Supplementary material for: Oral and Intravenous Fumonisin Exposure in Pigs—A Single-Dose Treatment Experiment Evaluating Toxicokinetics and Detoxification
Source: Toxins (Basel). 2018 Apr 5;10(4):150. doi: 10.3390/toxins10040150 (PMC5923316; doi:10.3390/toxins10040150)
Supplement: Supplementary file 1 [file toxins-10-00150-s001.pdf]

Supplementary Materials: Oral and Intravenous Fumonisin Exposure in Pigs—a Single-Dose Treatment Experiment Evaluating Toxicokinetics and Detoxification

Hanna Schertz, Jeannette Kluess, Jana Frahm, Dian Schatzmayr, Ilse Dohnal, Gerlinde Bichl, Heidi Schwartz-Zimmermann, Gerhard Breves and Sven Dänicke

Table S1. Composition of basal diet.

**Ingredients (g/kg)**	
Barley	745
Soybean meal	190
Soybean Oil	25
HCl-lysine	5
DL-methionine	3
L-threonine	2
L-tryptophan	1
Mineral and vitamin premix[1]	30
**Analysed composition**	
Dry matter (DM, %)	90.1
Crude protein (g/kg DM)	182.7
Crude fat (g/kg DM)	42.3
Crude fibre (g/kg DM)	43.5
Crude ash (g/kg DM)	65.9
Aflatoxin B1, B2, G1, G2 (LOD = 0.2 µg/kg)[2]	<LOD
Fumonisin B1 and B2 (LOD = 20 µg/kg)[2]	<LOD

[1]provided per kg premix: crude ash 90 %, Ca 24.5 %, P 6 %, Na 5.5 %, Mg 1 %, Fe 4,000 mg, Cu 1,000 mg, Mn 2,000 mg, Zn 4,000 mg, I 50 mg, Se 15 mg, Co 20 mg, vitamin A 400,000 I.U., vitamin D_3 40,000 I.U., vitamin E 1,200 mg, vitamin B_1 37.5 mg, vitamin B_2 100 mg, vitamin B_6 100 mg, vitamin B_{12} 750 mg, vitamin K_3 52.5 mg, nicotinic acid 500 mg, pantothenic acid 337.5 mg, choline chloride 5,000 mg.

[2]analysed by Romer Labs GmbH.
